# The Heart in the Transcatheter Intervention Era: Where Are We?

**DOI:** 10.3390/jcm11175173

**Published:** 2022-09-01

**Authors:** Ana Paula Tagliari, Maurizio Taramasso

**Affiliations:** 1 Post Graduate Program in Cardiology and Cardiovascular Science, Federal University of Rio Grande do Sul, Porto Alegre 90410-000, Brazil; 2 Cardiovascular Surgery Department, Hospital São Lucas da PUC/RS, Porto Alegre 90619-900, Brazil; 3 Cardiovascular Surgery Department, Hospital Mãe de Deus, Porto Alegre 90880-0481, Brazil; 4 HerzZentrum Hirslanden Zurich Clinic of Cardiac Surgery, 8008 Zurich, Switzerland

It is so exciting to imagine that the heart, once considered an untouchable organ, is now routinely approached by so many different techniques and with a wide array of invasiveness. However, this evolution, or better said, revolution, took a lot of time and a great effort from bright inventors to become a reality.

The first percutaneous balloon angioplasty in 1977 can be considered one of the landmarks for the development of transcatheter structural heart interventions, which was driven by the first-in-man balloon aortic valvuloplasty accomplished in 1986 by Alain Cribier [1] and by the first transcatheter implantation of an artificial aortic valve in pigs carried out by Henning Rud Andersen in 1992 [2]. Nonetheless, only 10 years later, Alain Cribier performed the first-in-man transcatheter aortic valve implantation (TAVI) using a balloon-expandable stented aortic valve device. That day, 16 April 2002, was a turning point in the history of Cardiac Surgery [3]. 

The patient was an inoperable 57-year-old male with severe aortic stenosis, who presented cardiogenic shock, with a left ventricle ejection fraction (LVEF) of only 12%. Balloon aortic valvuloplasty had failed, and performing the first TAVI seemed the only option to save his life. Since the patient had no transfemoral arterial access available, the physicians proceeded with a transvenous and transseptal approach. The operators’ main surprise was that, just a few minutes after the valve implantation, the patient’s blood pressure returned to normal, and his grey complexion turned into a healthy pink color. In Cribier’s own words: “we trusted in our idea, and our perseverance paid off. You can either give up, or you can find solutions, and that is what we did”. 

Undoubtedly, this first-in-man TAVI not only percutaneously treated that aortic valve stenosis but, most importantly, initiated the modern era of structural heart disease interventions. TAVI is now the standard-of-care treatment for inoperable and high-risk patients and a safe and effective option for those at intermediate and low risk [4,5,6,7,8,9,10,11]. It has been estimated that around 1.5 million patients in over 70 countries have had TAVI across these past 20 years. 

In the mitral valve arena, transcatheter edge-to-edge repair (TEER) can be considered in cases of chronic primary mitral regurgitation in severely symptomatic patients (NYHA functional class III or IV) with high or prohibitive surgical risk provided that the mitral valve anatomy is favorable and the patient life expectancy is at least 1 year (class of indication IIa B according to the ACC/AHA Guidelines; class of indication IIb B according to the ESC/EACTS Guidelines). In the context of chronic severe secondary mitral regurgitation related to left ventricular systolic dysfunction (LVEF < 50%) and persistent symptoms (NYHA functional class II, III, or IV), TEER is reasonable if appropriate anatomy, LVEF between 20% and 50%, left ventricular end-systolic diameter (LVESD) ≤ 70 mm, and pulmonary artery systolic pressure ≤ 70 mmHg (class of indication IIa B according to the ACC/AHA Guidelines) are present. According to the ESC/EACTS Guidelines, if a patient has severe secondary mitral regurgitation and concomitant coronary artery disease requiring treatment but is judged not appropriate for surgery, percutaneous coronary intervention, possibly followed by TTER (if persistent MR), should be considered (class of indication IIa C). On the other hand, if the patient had no concomitant coronary artery disease requiring treatment, TEER should be considered in selected symptomatic patients not eligible for surgery and fulfilling those criteria that suggest an increased chance of treatment response (class of indication IIa B). Lastly, for secondary tricuspid regurgitation, the transcatheter treatment may be considered in symptomatic inoperable patients (class of indication IIb C according to the ESC/EACTS Guidelines) [12,13]. 

Taking into consideration that every day new devices are developed and new indications for structural heart disease interventions are proposed, a high priority for the cardiovascular community must be to be engaged in this emerging area and to train the next generations of heart valve specialists, including surgeons, interventional and non-interventional cardiologists, heart failure and imaging specialists, anesthesiologists, geriatricians, nurse specialists, and researchers [14].

However, solid scientific evidence to support some of these new technological advancements is still lacking. Aiming to be part of this evidence generation process and present the most recent advances in transcatheter structural heart disease interventions, we provide this Special Issue. 

This Special Issue of the *Journal of Clinical Medicine* (JCM) entitled “Transcatheter Structural Heart Disease Interventions: Clinical Update” offers eight original articles and four review articles. Ten articles focus on the transcatheter aortic valve approach, discussing all the relevant issues related to this technique, such as balloon aortic valvoplasty [15]; TAVI indications and patient selection [16]; pre-procedural planning [17]; access routes (open or percutaneous vascular access) [18]; potential access-related complications [19]; TAVI outcomes compared with surgery [20]; challenges of surgery after TAVI failure [21]; post-TAVI prognostic factors [22]; potential benefits of cerebral embolic protection devices [23]; and the BASILICA technique to prevent coronary obstruction [24]. Three additional articles discuss the state of the art in transcatheter mitral valve replacement images [25]; atrial functional tricuspid regurgitation [26]; and the use of different diagnostic catheters for transradial coronary angiography [27].

In summary, the articles presented in this Special Issue cover a broad spectrum of transcatheter heart interventions guiding readers through the best evidence-based approach.
